# *Notes from the Field:* Transmission of Mpox to Nonsexual Close Contacts — Two U.S. Jurisdictions, May 1–July 31, 2022

**DOI:** 10.15585/mmwr.mm7250a3

**Published:** 2023-12-15

**Authors:** Ebony S. Thomas, Zachary J. Madewell, William L. Still, Julia Rowse, Brandon Shapiro, Emily Gately, Julie Shaffner, Anil T. Mangla, Jessica N. Ricaldi

**Affiliations:** ^1^CDC Multinational Monkeypox Response Team; ^2^District of Columbia Department of Public Health; ^3^Tennessee Department of Public Health; ^4^Division of State and Local Readiness, Center for Preparedness and Response, CDC.

During the 2022 multinational mpox outbreak, U.S. mpox cases primarily occurred among adult gay, bisexual, and other men who have sex with men (*1*). Among all cases, 94% of patients reported exposure through sexual or other intimate contact (*1*). Currently, little is known about less common nonsexual mpox transmission. A systematic review of mpox transmission in countries with endemic mpox in Central Africa found that the secondary attack rate among unvaccinated household contacts ranged from 0% to 11%, with a pooled estimate of 8% (*2*). A better understanding of the risk for nonsexual transmission during the current outbreak in countries where mpox is not endemic is important for developing and implementing future prevention and control strategies.

## Investigation and Outcomes

### Data Collection and Analysis

During August–September 2022, CDC requested that U.S. jurisdictions submit aggregate or deidentified individual-level data on the number of reported nonsexual contacts* of mpox patients with cases occurring during May 1–July 31. Most jurisdictions either reported no nonsexual contacts during the specified period or were unable to categorize contacts as nonsexual because of contact tracing limitations. Two jurisdictions, Tennessee and the District of Columbia (DC), reported aggregate data on the number of adult and pediatric nonsexual contacts identified during May 1–July 31, 2022. Data on the number of mpox patients’ nonsexual contacts interviewed and the exposure location were reported by DC. The exposure locations for nonsexual contacts of Tennessee patients were not reported. The secondary attack rate among nonsexual close contacts was defined as the percentage of nonsexual close contacts of mpox patients who became symptomatic within 21 days of exposure to the primary patient. Descriptive statistics were performed using SAS software (version 9.4; SAS Institute). This activity was reviewed by CDC, deemed not research, and was conducted consistent with applicable federal law and CDC policy.^†^

### Characteristics of Cases and Contacts

During May 1–July 31, a total of 278 mpox cases were reported by the two jurisdictions, and 662 nonsexual contacts of these patients were identified (average = 2.4 nonsexual contacts per patient) (Table). Among 563 nonsexual close contacts reported by DC, 162 (28.8%) were interviewed after exposure. The primary exposure settings for nonsexual contacts in DC were large gatherings (e.g., festivals) (230; 40.9%), unknown settings (119; 21.1%), place of employment (71; 12.6%), or home (44; 7.8%). Nine (secondary attack rate = 1.6%) nonsexual close contacts in DC experienced signs and symptoms within 21 days after exposure to the primary patient; five of these exposed persons who experienced signs and symptoms 21 days after exposure to the primary patient had received postexposure mpox vaccine before symptom onset.

**TABLE:** Number of mpox cases and nonsexual contacts — District of Columbia and Tennessee, May 1–July 31, 2022

Characteristic	Jurisdiction		
	Total	DC	Tennessee
Mpox patients, no.	**278**	252	26
Nonsexual close contacts, no. (average no. per primary patient)	**662 (2.4)**	563​ (2.2)	99 (3.8)
Nonsexual contacts per patient, mean	**2.4**	2.2	3.8
Nonsexual contacts interviewed ≥21 days after exposure, no. (%)*	**162 (24.5)**	162 (28.8)	​0 (—)
Reported nonsexual contact exposure setting, no. (%)*^,†^			
Large gatherings (e.g., festivals)	**NA**	230 (40.9)	NA
Unknown	**NA**	119 (21.1)	NA
Place of employment	**NA**	71 (12.6)	NA
Home	**NA**	44 (7.8)	NA
Secondary attack rate, no. (%) of secondary cases^§^	**10 (1.5)**	9 (1.6)	1 (1.0)

**Abbreviations:** DC = District of Columbia; NA = not available.

* Percentage of nonsexual contacts reported by primary patient.

^†^ This information is available for DC patients only.

^§^ Percentage of nonsexual contacts with symptom onset ≤21 days after exposure to the primary patient.

None of the 99 nonsexual contacts identified in Tennessee were interviewed ≥21 days after exposure. One of these contacts (secondary attack rate = 1.0%), who had received postexposure vaccination, experienced symptom onset within 21 days of exposure to the primary patient. Overall, a total of 10 persons who reported nonsexual close contact with an mpox patient experienced symptoms within 21 days after exposure (secondary attack rate = 1.5%).

### Limitations

The findings in this report are subject to at least six limitations. First, age-specific information was not reported for nonsexual contacts; therefore, this report cannot distinguish between pediatric and adult nonsexual contacts. Second, fewer than one half of nonsexual contacts in one jurisdiction were interviewed after exposure, which might have resulted in underreporting of secondary cases. Third, type of contact was self-reported, which might be subject to recall or social desirability bias. Fourth, data were incomplete for many nonsexual close contacts. Fifth, the first 2 months (May–June) of the 3-month study period included the period before availability of postexposure prophylaxis had been expanded, and mpox cases might have overidentified close contacts to facilitate receipt of postexposure mpox vaccination, potentially artificially inflating close contact numbers. Finally, because this investigation did not collect mpox laboratory test results for nonsexual close contacts who became symptomatic 21 days after exposure to the primary case, the secondary attack rate might be inflated.

## Preliminary Conclusions and Actions

Although sexual or intimate contact was the primary mode of transmission in the 2022 multinational mpox outbreak, limited nonsexual transmission also occurred. The secondary attack rate reported from this investigation is consistent with that reported among nonsexual contacts in regions with endemic mpox (*2*). Monitoring of nonsexual contacts for mpox signs and symptoms is warranted after a known exposure to an mpox patient. Nonsexual contacts of mpox patients should be educated about prevention methods and steps to take should they experience signs and symptoms after exposure (*3*). Ongoing collection and analysis of data from nonsexual close contacts by state and local health departments and CDC, including factors associated with an increased risk for infection and behaviors that increase the risk for transmission, can help guide development and implementation of recommendations.

## Footnotes

* Nonsexual contact was defined as exposure to respiratory droplets from someone with mpox, direct contact with contaminated items, or nonintimate skin-to-skin contact with an mpox patient, and no reported sexual contact with an mpox patient during the preceding 3 weeks.

† 45 C.F.R. part 46.102(l)(2), 21 C.F.R. part 56; 42 U.S.C. Sect. 241(d); 5 U.S.C. Sect. 552a; 44 U.S.C. Sect. 3501 et seq.
